# Neonatal Hypothermia and Associated Factors among Newborns Admitted in the Neonatal Intensive Care Unit of Dessie Referral Hospital, Amhara Region, Northeast Ethiopia

**DOI:** 10.1155/2020/3013427

**Published:** 2020-09-14

**Authors:** Yibeltal Asmamaw Yitayew, Endashaw Belayhun Aitaye, Helina Wondimu Lechissa, Lubaba Oumer Gebeyehu

**Affiliations:** Department of Pediatrics and Child Health Nursing, College of Medicine and Health Science, Wollo University, Ethiopia

## Abstract

**Introduction:**

Neonatal hypothermia is the reduction in the body temperature of the newborn (less than 36.5°C). It is a global problem in neonates born both at hospitals and homes, but it showed a higher prevalence in developing countries (>90%). Although hypothermia is rarely a direct cause of death, it contributes to a substantial proportion of neonatal mortality globally.

**Objective:**

To assess neonatal hypothermia and associated factors among newborns admitted in the NICU of Dessie Referral Hospital.

**Methods and Materials:**

An institution-based cross-sectional study was conducted from March 15 to May 30, 2018. The data was collected from the mother and the chart of the newborn using a semistructured questionnaire. Data were cleaned, coded, and entered in EPI-info version 7.1.2.0 then exported to Statistical Package for Social Sciences (SPSS) version 20 software for analysis. Descriptive statistics were used to summarize the data. Bivariate and multivariate logistic regression and crude and adjusted odds ratio with their 95% confidence interval were computed. Finally, *p* value < 0.05 was used to identify variables that had a significant association with neonatal hypothermia.

**Result:**

The proportion of neonatal hypothermia in the study area was 66.8%. Preterm delivery (AOR = 2.6, 95% CI: 1.1, 6.2), no skin-to-skin contact within 1 hour of delivery (AOR = 3.0, 95% CI: 1.3, 7.8), delivered at night time (AOR = 2.0, 95% CI: 1.02, 4.0), and neonates who had resuscitation (AOR = 2.9, 95% CI: 1.1, 7.2) showed significant association with neonatal hypothermia.

**Conclusion:**

In this study, the proportion of hypothermia was high. Preterm delivery, no skin-to-skin contact within 1 hour, night-time delivery, and having resuscitation were significantly associated with neonatal hypothermia. Therefore, special attention is needed for the thermal care of preterm neonates and neonates delivered at night time. Furthermore, there should be strict adherence to cost-effective thermal care recommendations like warm resuscitation and skin-to-skin contact.

## 1. Introduction

Globally, 2.5 million children died in the first months of life in 2017, which accounts for 47% of all under-five child deaths [1]. Most neonatal deaths (99%) arise in low and middle-income countries, and the leading causes of death include prematurity, birth asphyxia, infection, and birth defects [2]. Although hypothermia is rarely a direct cause of death, it contributes to a substantial proportion of neonatal mortality globally, mostly as a comorbidity [3]. A high prevalence of neonatal hypothermia has been reported from countries with the highest burden of neonatal mortality [4]. Therefore, reducing the prevalence of neonatal hypothermia in low-resource communities has a significant contribution to reducing the global burden of neonatal deaths [4, 5].

Neonatal hypothermia is a progressive reduction in the axillary temperature of the newborn (temperature < 36.5°C). It is categorized as mild hypothermia (36°C–36.4°C), moderate hypothermia (32°C–35.9°C), and severe hypothermia (<32°C) [6]. Neonates are prone to rapid heat loss and consequent hypothermia because of the large surface area-to-body mass ratio, decreased subcutaneous fat, immature skin, high body water content, poorly developed metabolic mechanism, and altered skin blood flow [7]. Hypothermic neonates had a higher risk of developing hypoglycemia, respiratory distress syndrome, jaundice, and metabolic acidosis [8].

Although neonatal hypothermia is a global problem that occurs in neonates born both at hospitals (32%-85%) and homes (11%-92%), it showed higher prevalence in developing countries (>90%) [3, 9]. Similarly, different studies conducted in Ethiopia showed that the prevalence of neonatal hypothermia ranges from 53% to 69.8% [3, 10, 11].

Physiological, environmental, and behavioral risk factors predispose the newborn infants for neonatal hypothermia [4]. Thermal care is the major component of essential newborn care package included in the World Health Organization (WHO) to be applied universally for all babies to decrease neonatal mortality [12]. In Ethiopia, the principle of skin-to-skin contact and initiating breastfeeding within the first hour after birth was the common practice to prevent hypothermia, which is also recommended by the WHO [13]. Besides this intervention, neonatal hypothermia is still high in Ethiopia [3, 10, 11].

As a result, identifying different factors that had an association with neonatal hypothermia is valuable to design an appropriate intervention strategy to decrease neonatal hypothermia as well as to improve the quality of newborn care. So, this study was aimed to assess neonatal hypothermia and associated factors among newborns admitted in the NICU of Dessie Referral Hospital.

## 2. Materials and Method

### 2.1. Study Design, Setting, and Period

A hospital-based cross-sectional study design was conducted from March 15 to May 30, 2018, in the NICU of Dessie Referral Hospital. Dessie town is located in South Wollo Zone of Amhara Regional State that is 401 km away from Addis Ababa, the capital city of Ethiopia, and 480 km from Bahir Dar, the capital city of Amhara Regional State. Dessie Referral Hospital deliver health care services for patients coming from different areas, including the Afar region. In 2017, over five thousand mothers gained antenatal, delivery, and postnatal care services, and there were 1868 neonatal admissions in the NICU.

### 2.2. Source and Study Population

All neonates with their mothers admitted in the NICU of Dessie Referral Hospital were the source populations, and the study populations were randomly selected neonates with their mothers admitted in the NICU of Dessie Referral Hospital from March 15 to May 30, 2018.

### 2.3. Exclusion Criteria

Neonates whose mother was not present during the study period were excluded.

### 2.4. Sample Size Determination and Sampling Procedure

The sample size was determined using a single population proportion formula with the assumption of 64% (*p* = 0.64) proportion of neonatal hypothermia taken from the study conducted in Addis Ababa University, with a 95% confidence level and 5% margin of error [10]. The initial sample size was calculated to be 354. The source population in the data collection period was estimated to be 415, which is less than 10,000. As a result, the following correction formula was used to calculate the final sample size:

(1) $$\begin{array}{c} n_{f}=\frac{n_{i}}{1+\left(n_{i}/N\right)}=\frac{354}{1+\left(354/415\right)}=191, \end{array}$$

where *n*_f_ is the final sample size, *n*_i_ is the initial sample size, and *N* is the total estimated neonatal admission during the data collection time.

By considering a 10% nonresponse rate of participants, the final sample size was 210. A systematic random sampling technique (*k* = 2) was used to select the study participants based on their admission and registration number.

### 2.5. Data Collection Tool, Procedure, and Quality Control

The data were collected from the mother and the chart of the newborn using a semistructured questionnaire that was adopted and modified from a study conducted in Addis Ababa, Gondar, Nigeria, and Uganda [10, 11, 14, 15]. The axillary temperature of the newborn was measured at the point of admission by using a digital thermometer (model-MT-101) that had a measurement accuracy of ±0.1°C for the temperature range of 35.5°C–42.0°C and ±0.2°C for the temperature range of 32.0°C–35.5°C or above 42.0°C [10, 16]. The data were collected by three BSc neonatal nurses who were working in the NICU and supervised by one MSc pediatric and child health nurse professional. Two days of training was given for data collectors, and the pretest was conducted in 5% of the final sample size in Akasta Hospital. Moreover, the supervisor and principal investigators conducted regular supervision and checked the data for completeness.

### 2.6. Data Processing, Analysis, and Presentation

All field questionnaires were checked for completeness, consistency, and accuracy. The data were entered into EPI info (version 7.1.2.0) then exported to SPSS (Statistical Package for Social Sciences, version 22) for data analysis. Descriptive statistics (frequency table, pie chart, and bar graph) were used to summarize the data. Bivariate logistic regression was used to assess the association of independent variables with the outcome variable. Variables found to have *p* value < 0.2 in bivariate logistic regression were further analyzed using multivariate logistic regression. Odds ratio (OR) with 95% CI was used as a measure of association, and variables that had a *p* value less than 0.05 in the multivariate logistic regression were considered as a significantly associated variable.

### 2.7. Operational Definition and Definition of Terms

Hypothermia: an axillary temperature of less than 36.5°C.

Mild hypothermia (cold stress): an axillary temperature of 36.0°C-36.4°C.

Moderate hypothermia: an axillary temperature of 32.0°C to 35.9°C.

Severe hypothermia: an axillary temperature of <32.0°C.

Nonhypothermic: an axillary temperature of ≥36.5°C.

Neonate: an infant under 28 days of age.

Admission temperature: the first temperature obtained from the neonate at admission in the NICU.

Inborn: a newborn delivered from the study hospital (Dessie Referral Hospital).

Outborn: a newborn delivered other than the study hospital.

## 3. Result

### 3.1. Sociodemographic Characteristics

A total of 202 neonates with their mother admitted in the NICU were included in the study, resulting in a 96.2% response rate. The majority (72.3%) of mothers were aged 20-29 years (mean age and SD = 25.88 ± 4.86 years). One hundred fifty-four (76.2%) and 65 (32.2%) respondents were from urban residents and attained primary education, respectively. Seventy-five (37.1%) were housewives in occupation and one hundred eighteen (58.4%) respondents had monthly income ≥ 2500 ETB (mean and SD = 2944 ± 1731 ETB) (Table 1).

### 3.2. Neonatal Characteristics

Out of 202 neonates, 111 (55%) were male, and 146 (72.3%) had gestational age ≥ 37 weeks (mean and SD = 37.4 ± 2.5 weeks) at delivery. Most of the neonates 124 (61.4%) had birth weight ≥ 2500 g (mean and SD = 2580.9 g ± 732.8 gram), and 44 (21.8%) neonates received CPR. One hundred sixty-one (79.7%) had skin-to-skin contact immediately after birth, and 175 (86.6%) had late initiation of breastfeeding (Table 2).

### 3.3. Obstetric and Environmental Factors

The majority of the pregnancies 192 (95%) were a singleton, 116 (57.4%) of mothers were primiparous, and 126 (62.4%) neonates were delivered in SVD. Most of the participants 179 (88.6%) had no obstetric complications, and 118 (58.4%) mothers delivered during night time. Nearly all neonates 199 (98.5%) were admitted in the NICU at a room temperature of ≥25°C (mean and SD = 27.2 ± 1.3°C) (Table 3).

### 3.4. Proportion of Hypothermia

**Figure 1:**
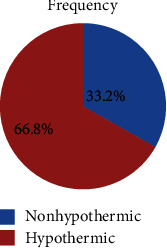
Distribution of neonates in relation to the presence of hypothermia in Dessie Referral Hospital, Northeast Ethiopia, 2018 (*N* = 202).

**Figure 2:**
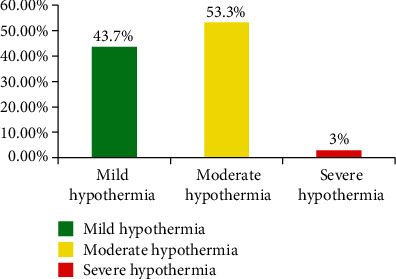
Distribution of hypothermic neonates in relation to degree of hypothermia in Dessie Referral Hospital, Northeast Ethiopia, 2018 (*N* = 202).

**Figure 3:**
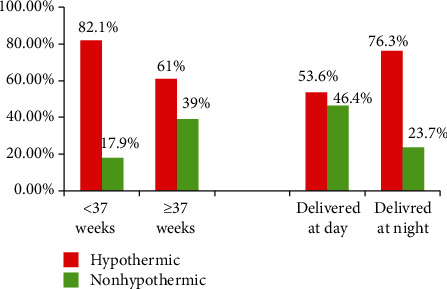
Comparison of hypothermia with gestational age and time of delivery in Dessie Referral Hospital, Northeast Ethiopia, 2018 (*N* = 202).

Out of the total neonates, 135 (66.8%) were hypothermic at the time of admission in the NICU, and the mean and SD of axillary temperature were 35.8 ± 1.3°C. From those hypothermic neonates, the majority (53.3%) had moderate degree hypothermia (Figures 1–3).

### 3.5. Bivariate and Multivariate Analyses of Factors Associated with Neonatal Hypothermia

In the bivariate analysis, birth weight, gestational age, skin-to-skin contact with their mother immediately after delivery, initiation of breastfeeding within 1 hour, bathing within 24 hours, time of delivery, and undergoing CPR procedure showed association with the occurrence of neonatal hypothermia (*p* value < 0.2) (Table 4).

Variables, which had a *p* value of <0.2 in the bivariate analysis, were further analyzed using multivariate logistic regression. The result of this analysis showed that preterm delivery, no skin-to-skin contact with their mother immediately after birth, time of delivery, and undergoing CPR procedure were significantly associated variables.

Preterm neonates were almost 2.6 times more likely to have hypothermia compared with term neonates (*p* = 0.03, 95% CI: 1.1-6.2). Neonates who had no skin-to-skin contact with their mother immediately after delivery were 3.1 times more likely to be hypothermic when compared to those who have skin-to-skin contact (*p* = 0.041, 95% CI: 1.3-7.8). Similarly, neonates delivered at night time were 2 times more likely to have hypothermia compared with neonates delivered at day time (*p* = 0.045, 95% CI: 1.02-4). Newborns who had resuscitation at birth (CPR) were 2.9 times more likely to be hypothermic when compared with those who had no resuscitation (*p* = 0.024, 95% CI: 1.1-7.2) (Table 5).

## 4. Discussion

In this study, the proportion of hypothermia was 66.8% (95%CI = 60.4%–73.7%). The finding of this study was comparable with studies conducted in Nigeria (62% and 67.6%), Gondar teaching and referral hospital (68.9%), and governmental hospitals in Addis Ababa (64%) [10, 11, 15, 17]. However, this finding was higher than studies conducted in Iran (53.3%), Bangladesh (34%), India (43%), and South Africa (21%) [18–21] but lower than the study conducted in Nepal [9]. The possible reason for this discrepancy might be the variation in the weather condition of study areas since Dessie is a cold town which has an altitude of 2470 meter [22]. Additionally, differences in study design, study setting, temperature measurement site, and cultural and economic factors may contribute to the differences.

In this study, gestational age was significantly associated with neonatal hypothermia. The odds of neonatal hypothermia were 2.6 times higher in preterm than term neonates. The possible reason for this finding might be preterm neonates have a large surface area-to-body mass, minimal subcutaneous fat stores, poor clinical status, low tone, and limited capacity to generate heat from fat stores [23]. This finding is in line with studies conducted in Addis Ababa (AOR = 4.81), Tigray (AOR = 3.7), and Iran (AOR = 1.73) [10, 18, 24].

Neonates who had no skin-to-skin contact within 1 hour after delivery had a 3.1 times higher odds of hypothermia compared to those who had skin-to-skin contact. Skin-to-skin contact enables the newborn to achieve and maintain thermal control, better temperature gain, and lesser morbidity [25, 26]. This finding is comparable to the studies conducted in Iran (AOR = 3.27), Addis Ababa (AOR = 4.39), Tigray (AOR = 6.2), and Gondar (AOR = 2.82) [10, 11, 24].

Delivery time was the other variable that showed a significant association with neonatal hypothermia. Neonates delivered at night time were 2 times more likely to develop hypothermia than neonates delivered at day time. These will be due to the temperature difference at night and day time. When there is no provision of added warmth during cold nights, the newborn infant is at risk of becoming hypothermic [27]. Additionally, in this study, the majority (58.4%) of newborns were delivered at night, where there is a limited number of working staffs in the labor ward. This finding is in line with studies conducted in Gondar (AOR = 6.6) and Tigray (AOR = 6.25) [11, 24].

Another variable that showed significant association in this study was neonatal resuscitation. The odds of hypothermia were 2.9 times higher in neonates having CPR than those who had not. Heat loss is a particular problem at resuscitation. Keeping infants sufficiently warm immediately after birth, especially during resuscitation, is difficult [28, 29]. A similar finding was reported from studies conducted in Addis Ababa (AOR = 3.65), Bangladesh (AOR = 2.43), and Iran (AOR = 1.91) [10, 18, 19].

### 4.1. Limitation of the Study

This study was not conducted in multiple health facilities including private hospitals, and it did not incorporate a qualitative method to address cultural and behavioral factors that will affect neonatal hypothermia. The effect of seasonal variations was not included because of a short data collection period.

## 5. Conclusion

In this study, the proportion of hypothermia was high (66.8%). Being preterm, no skin-to-skin contact with their mother, night-time delivery, and neonatal resuscitation had a significant association with neonatal hypothermia. Therefore, health care providers should have special attention for the thermal care of preterm neonates and newborns delivered at night time. Additionally, there should be strict adherence to the WHO recommendation of thermal care for newborns including warm resuscitation and skin-to-skin contact immediately after delivery.

## Figures and Tables

**Table 1:** Sociodemographic characteristics of mothers in Dessie Referral Hospital, Northeast Ethiopia, 2018 (*N* = 202).

Variables	Category	Frequency (%)		
		Hypothermic	Nonhypothermic	Total
Age of the mother (years)	<20	8 (3.96%)	7 (3.47%)	15 (7.4%)
	20-29	101 (50%)	45 (22.28%)	146 (72.3%)
	30-39	22 (10.9%)	14 (6.9%)	36 (17.8%)
	≥40	4 (1.98%)	1 (0.5%)	5 (2.5%)
Ethnicity	Amhara	127 (62.9%)	65 (32.2%)	192 (95%)
	Tigray	5 (2.5%)	1 (0.5%)	6 (3%)
	Oromo	3 (1.5%)	1 (0.5%)	4 (2%)
Religion	Orthodox	41 (20.3%)	24 (11.9%)	65 (32.2%)
	Protestant	13 (6.4%)	1 (0.5%)	14 (6.9%)
	Muslim	81 (40.1%)	42 (20.8%)	123 (60.9%)
Residence	Urban	102 (50.5%)	52 (25.7%)	154 (76.2%)
	Rural	33 (16.4%)	15 (7.4%)	48 (23.8%)
Educational status	Unable to read and write	24 (11.9%)	13 (6.4%)	37 (18.3%)
	Primary education	41 (20.3%)	24 (11.9%)	65 (32.2%)
	Secondary education	35 (17.3%)	17 (8.4%)	52 (25.7%)
	Diploma and above	35 (17.4%)	13 (6.4%)	48 (23.8%)
Maternal occupation	Housewife	49 (24.2%)	26 (12.9%)	75 (37.1%)
	Governmental employee	26 (12.9%)	10 (4.9%)	36 (17.8%)
	Self-employed	30 (14.9%)	14 (6.9%)	44 (21.8%)
	Student	8 (4%)	2 (1%)	10 (5%)
	Farmer	22 (10.9%)	15 (7.4%)	37 (18.3%)
Family monthly income	<1000	12 (5.9%)	4 (2%)	16 (7.9%)
	1000-1499	29 (14.4%)	10 (5%)	39 (19.4%)
	1500-2499	15 (7.4%)	14 (6.9%)	29 (14.3%)
	≥2500	79 (39.1%)	39 (19.3%)	118 (58.4%)

**Table 2:** Neonatal factors of the study participants in Dessie Referral Hospital, Northeast Ethiopia, 2018 (*N* = 202).

Variables	Category	Frequency (%)		
		Hypothermic	Nonhypothermic	Total
Sex of neonate	Male	75 (37.1%)	36 (17.8%)	111 (54.9%)
	Female	60 (29.7%)	31 (15.3%)	91 (45%)
Age of neonate (hours)	<24	79 (39.1%)	45 (22.3%)	124 (61.4%)
	≥24	56 (27.7%)	22 (10.9%)	78 (38.6%)
Birth weight (grams)	<2500	59 (29.2%)	19 (9.4%)	78 (38.6%)
	≥2500	76 (37.6%)	48 (23.8%)	124 (61.4%)
Gestational age (weeks)	<37	46 (22.8%)	10 (4.9%)	56 (27.7%)
	≥37	89 (44.1%)	57 (28.2%)	146 (72.3%)
Bathing before 24 hour	Yes	19 (9.4%)	5 (2.5%)	24 (11.9%)
	No	116 (57.4%)	62 (30.7%)	178 (88.1%)
Start breastfeeding within 1 hour after birth	Yes	15 (7.4%)	12 (6%)	27 (13.4%)
	No	120 (59.4%)	55 (27.2%)	175 (86.6%)
Skin-to-skin contact immediately after birth	Yes	17 (8.4%)	24 (11.9%)	41 (20.3%)
	No	118 (58.4%)	43 (21.3%)	161 (79.7%)
Received CPR	Yes	37 (18.3%)	7 (3.5%)	44 (21.8%)
	No	98 (48.5%)	60 (29.7%)	158 (78.2%)

**Table 3:** Obstetric and environmental factors of the study participants in Dessie Referral Hospital, Northeast Ethiopia, 2018 (*N* = 202).

Variables	Category	Frequency (%)		
		Hypothermic	Nonhypothermic	Total
Parity	Primiparous	79 (39.1%)	37 (18.3%)	116 (57.4%)
	Multiparous	56 (27.7%)	30 (14.9%)	86 (42.6%)
Pregnancy type	Single	128 (63.3%)	64 (31.7%)	192 (95%)
	Twin	7 (3.5%)	1 (0.5%)	8 (4%)
	Triple	—	2 (1%)	2 (1%)
Complication during pregnancy	Yes	18 (8.9%)	5 (2.5%)	23 (11.4%)
	No	117 (57.9%)	62 (30.7%)	179 (88.6%)
Mode of delivery	SVD	82 (40.6%)	44 (21.8%)	126 (62.4%)
	Instrumental	27 (13.4%)	15 (7.4%)	42 (20.8%)
	CS	26 (12.9%)	8 (4%)	34 (16.8%)
Place of delivery	Inborn	58 (28.7%)	27 (13.4%)	85 (42.1%)
	Outborn	77 (38.1%)	40 (19.8%)	117 (57.9%)
Setting for outborn delivery	Other hospital	19 (16.2%)	14 (12%)	33 (28.2%)
	Health center	50 (42.7%)	13 (11.1%)	63 (53.8%)
	Private institution	—	6 (5.1%)	6 (5.1%)
	At home	8 (6.8%)	7 (6%)	15 (12.8%)
Time of delivery	Day	45 (22.3%)	39 (19.3%)	84 (41.6%)
	Night	90 (44.6%)	28 (13.8%)	118 (58.4%)
Room temperature of the NICU	<25°C	1 (0.5%)	2 (1%)	3 (1.5%)
	≥25°C	134 (66.3%)	65 (32.2%)	199 (98.5%)

**Table 4:** Bivariate logistic regression analysis of factors associated with neonatal hypothermia in the NICU of Dessie Referral Hospital, Ethiopia, 2018 (*N* = 202).

Variables		Hypothermia		COR (95% CI)	*p* value
		Yes	No		
Birth weight (grams)	<2500	59 (29.2%)	19 (9.4%)	2 (1.04, 3.7)	0.036
	≥2500	76 (37.6%)	48 (23.8%)	1	
GA (weeks)	<37	46 (22.8%)	10 (5%)	2.9 (1.4, 6.3)	0.005
	≥37	89 (44%)	57 (28.2%)	1	
Skin-to-skin contact	Yes	17 (8.4%)	24 (11.9%)	1	0.0001
	No	118 (58.4%)	43 (21.3%)	3.9 (1.9, 7.9)	
Initiation of BF within 1 hr	Yes	15 (7.4%)	12 (6%)	1	0.185
	No	120 (59.4%)	55 (27.2%)	1.7 (0.8, 4)	
Bathing within 24 hours	Yes	19 (9.4%)	5 (2.5%)	2 (0.7, 5.7)	0.18
	No	116 (57.4%)	62 (30.7%)	1	
Time of delivery	Day	45 (22.3%)	39 (19.3%)	1	0.001
	Night	90 (44.6%)	28 (13.8%)	2.8 (1.5, 5)	
CPR	Yes	37 (18.3%)	7 (3.5%)	3.2 (1.4, 7.7)	0.008
	No	98 (48.5%)	60 (29.7%)	1	

**Table 5:** Multivariate logistic regression analysis of factors associated with neonatal hypothermia in the NICU of Dessie Referral Hospital, Ethiopia, 2018 (*N* = 202).

Variables		Hypothermia		COR (95% CI)	AOR (95% CI)	*p* value
		Yes	No			
Birth weight (grams)	<2500	59 (29.2%)	19 (9.4%)	1.96 (1.04, 3.7)	1 (0.5-2.3)	0.9
	≥2500	76 (37.6%)	48 (23.8%)	1	1	
GA (weeks)	<37w	46 (22.8%)	10 (5%)	2.95 (1.4, 6.3)	**2.60 (1.1-6.2)** ^∗^	**0.03**
	≥37w	89 (44%)	57 (28.2%)	1	1	
Skin-to-skin contact	Yes	17 (8.4%)	24 (11.9%)	1	1	**0.014**
	No	118 (58.4%)	43 (21.3%)	3.87 (1.9, 7.9)	**3.14 (1.3-7.8)** ^∗^	
Initiation of BF within 1 hr	Yes	15(7.4%)	12 (6%)	1	1	0.39
	No	120 (59.4%)	55 (27.2%)	1.75 (0.8, 4)	1.6 (0.5-4.8)	
Bathing within 24 hours	Yes	19 (9.4%)	5 (2.5%)	2.03 (0.7, 5.7)	1.9 (0.6-5.6)	0.26
	No	116 (57.4%)	62 (30.7%)	1	1	
Time of delivery	Day	45 (22.3%)	39 (19.3%)	1	1	**0.045**
	Night	90 (44.6%)	28 (13.8%)	2.79 (1.5, 5)	**2.03 (1.02-4)** ^∗^	
Undergoing CPR	Yes	37 (18.3%)	7 (3.5%)	3.24 (1.4, 7.7)	**2.88 (1.1-7.2)** ^∗^	**0.024**
	No	98 (48.5%)	60 (29.7%)	1	1	

∗ = statically significant at *p* < 0.05 with 95% CI.

## Data Availability

The data used to support the findings of this study are available from the corresponding author upon request.
